# Study of High Sensitive C-Reactive Protein (HS-CRP) After Cardiac Rehabilitation Program in Patients Undergoing Isolated CABG

**Published:** 2016-12

**Authors:** Adel Johari Moghadam, Saied Azizinejad

**Affiliations:** Department of Cardiology, AJA University of Medical Sciences, Tehran, Iran

**Keywords:** High sensitive C - reactive protein (HS-CRP), Cardiac rehabilitation program, Coronary artery, Surgery, Patient

## Abstract

**Introduction:**

Although cardiac rehabilitation is known as a tool to reduce the overall risk of cardiovascular complications, its specific role in the reduction of hs-CRP as a marker of inflammation and a proven marker of cardiovascular risk needs further investigation.

**Aim:**

The present study aims at elucidating the effects of a full course of conventional cardiac rehabilitation program for the period of eight weeks, on the levels of hs-CRP in patients who underwent isolated coronary artery bypass surgery.

**Material and Methods:**

In this case study, 30 consecutive patients who underwent isolated coronary artery bypass surgery (isolated CABGS), and a full 8-week cardiac rehabilitation program in Tehran Heart Center, were investigated. A group of 30 similar patients, who enrolled in the same period of rehabilitation program but did not participate in practice, was considered as a control group. Serum levels of hs-CRP in both groups were measured retrospectively and in similar days before the start of rehabilitation program and at the end of it (or 8 weeks after initial registration for the control group).

**Results:**

Levels of hs-CRP in the rehabilitation group and control group were 5.9 7.7 and 6.3 6.9 respectively before start of the program which was not statistically meaningful (*P*-Value = 0.833). However, after the program, level of hs-CRP in the two tested groups changed to 2.3 5.1 and 5.7 6.1 respectively which showed a meaningful correlation (*P*-Value = 0.023). These results also showed that decrease in hs-CRP level in the rehabilitated group but not in the control group was statistically meaningful (with *P*-Value of 0.037 and 0.0723 respectively).

**Conclusion:**

In patients undergoing coronary bypass surgery, participating in a full course of cardiac rehabilitation for 8 weeks has resulted in a significant reduction in hs-CRP levels as a marker of cardiovascular risk.

## INTRODUCTION

To reduce the risk of coronary heart disease and cardiac mortality after myocardial ischemia, it is quite necessary to consider secondary prevention. Therefore improving physical activity and controlling risk factors such as inflammatory markers of these disorders is highly recommended (1).

The effect of cardiac rehabilitation programs to reduce mortality, improve physical capacity, control of blood pressure, control of vital signs and symptoms of heart or lung has been proven in many studies (2, 3). However, the effect of cardiac rehabilitation program on inflammatory markers in this disorder is not well known.

On the other hand, recent studies have shown that regular exercise significantly decreases serum levels of inflammatory markers (4, 5). This result could be an indication of the likely impact of the regular program of cardiac rehabilitation on the control of inflammatory processes that generate atherothrombosis in patients who suffer from cardiac disorders. Hence, determination of serum levels of hs-CRP index after cardiac rehabilitation program in patients undergoing isolated coronary artery bypass surgery could give a better understanding of recurrence.

C-reactive protein (CRP) was first discovered in 1930 through a reaction with the somatic C polysaccharide of Streptococcus pneumonia in patients infected with pneumonia (6). Its correlation with coronary heart disease (CHD) was reported more than 60 years later in 1990 (7).

CRP has been extensively investigated and has long plasma half-life of 19 h. It is also quite easy to test with a standardized assay (8). CRP is an acute-phase protein and nonspeciﬁc marker of inﬂammation. The main production site of CRP is hepatocytes and it is consistent of five identical subunits. CRP is being secreted in response to several cytokines (9). Interleukin (IL)-6, one of the most potent drivers of CRP production, is released from activated leukocytes in response to infection or trauma and from vascular smooth muscle cells in response to atherosclerosis.

CRP directly binds highly atherogenic oxidized low-density lipoprotein cholesterol (LDL-C) and is present within lipid-laden plaques (10, 11). The possible mechanistic role of CRP in plaque deposition is highly complex, exerting proatherogenic effects in many cells involved in atherosclerosis (12). CRP may facilitate monocyte adhesion and transmigration into the vessel wall (13). Moreover, M1 macrophage polarization, catalyzed by CRP, is a proinﬂammatory trigger in plaque deposition, prompting macrophage inﬁltration of both fat tissue and atherosclerotics (14).

In addition to triggering immunity in plaque deposition, in vitro studies have also shown relation among CRP, inhibition of endothelial nitric oxide synthase, and debilitated vasoreactivity (15, 16). Monomeric CRP is stimulated by platelet activation and has prothrombotic and inflammatory properties of its own (17). Monomeric CRP has also been found in plaques, particularly in regions of monocyte-mediated inflammatory activity, and within lipid microdomains of endothelial cells (18).

Correlation between hs-CRPand risks of CVD has been described in many studies (19). The Multiple Risk Factor Intervention Trial was the first of many primary prevention, prospective epidemiological studies to show a strong relationship between levels of hs-CRP and mortality from CHD in high-risk middle aged men (7). A similar association between elevated hs-CRP levels and subsequent rate of mass index and susceptibility for stroke was found in an investigation of apparently healthy men (20).

The present study aims at elucidating the effects of a full course of conventional cardiac rehabilitation program for the period of eight weeks, on the levels of hs-CRP in patients who underwent isolated coronary artery bypass surgery.

## MATERIALS AND METHODS

All of patients who underwent CABG and were candidate for the rehabilitation program were included in this study. Those patients who were not able to participate in the program (unstable angina, unstable arrhythmia…) or were unwilling to do so were excluded from the study. All patients have signed consent forms before entering the study.

5 ml of blood samples from fasting patients 12 L 14 an hour had passed, they were taken to measure serum hs-CRP was sent to the laboratory. The index level was measured using a laser-based fluorescent analytic system. Measurement was done in two periods of time; at the time of administration for the program and at the end of rehabilitation period. The study was conducted at the Tehran Heart Center. Rehabilitation program consisted of 24 sessions over a period of 8 weeks.

The duration of each session was about thirty minutes and was performed as follow:

1. Warm-up (3-5 min
2. Aerobic Exercise (32-45 min)
3. Cool-down (3-5 min)

Aerobic exercise has a certain protocol and is set according to ‘Maximum Heart Rate’ and ‘Risk Stratification’. In addition, at the beginning of the project, all patients underwent the training program includes counseling and nutrition. The normal distribution of continuous variables was checked with paired t-test.

## RESULTS

Rehabilitation and control groups were similar in demographic characteristics. Also there was no significant difference in risk factors of heart disease between two groups (Table 1).

Table 2 represents results of comparison of HS-CRP level between two tested groups. Levels of HS-CRP in the rehabilitation group and control group were 5.9 7.7 and 6.3 6.9 respectively before start of the program which was not statistically meaningful (*P*-Value = 0.833). However, after the program, level of HS-CRP in the two tested groups changed to 2.3 5.1 and 5.7 6.1 respectively which showed a meaningful correlation (*P*-Value = 0.023). These results also showed that decrease in HS-CRP level in the rehabilitated group but not in the control group was statistically meaningful (with *P*-Value of 0.037 and 0.0723 respectively).

**Table 1:** Basic characteristics of the rehabilitation and control groups

Data	Rehabilitation Group (n=30) (%)	Control Group (n=30) (%)	*P*-Value

Male Gender	26 (87.6)	24 (80.0)	0.835
Age (Years)	59.8 ± 9.1	62.2 ± 9.0	0.309
BMI (kg/m^2^)	27.3 ± 3.9	26.8 ± 3.5	0.603
Smoker	10 (33.4)	7 (23.7)	0.520
Diabetes Mellitus	9 (30)	8 (26.7)	0.830
Hyperlipidemia	15 (50.0)	17 (56.6)	0.775
Hypertension	13 (43.3)	14 (46.7)	0.873
Family history of CAD	15 (50.9)	17 (56.6)	0.775
Renal Failure	1 (3.3)	0 (0.0)	0.999
Ejection Fraction	48.4 ± 8.2	48.1 ± 8.7	0.891
Function Class I	25 (83.3)	26 (86.7)	0.918
Function Class II	5 (16.7)	4 (13.3)	0.756

**Table 2:** HS-CRP level in rehabilitation and control groups before and after rehabilitation program

	Before Rehabilitation	After Rehabilitation	*P*-Value

Rehabilitation Group	5.9 ± 7.7	2.3 ± 5.1	0.037
Control Group	6.3 ± 6.9	5.7 ± 6.1	0.723
*P*-Value	0.833	0.023	

Categorical data are expressed as number (%), continuous variables as mean and standard deviation (mean ± SD).

## DISCUSSION

Inflammation plays an important role in the development of atherosclerotic plaque. The most important inflammatory marker known for ischemic heart disease is hs-CRP. This indicator plays an important role in the prognosis of patients with cardiac ischemia. The aim of this study was to evaluate the impact of cardiac rehabilitation on the serum index of this factor in the patients after coronary bypass surgery.

Our results showed that participation in cardiac rehabilitation program could reduce serum levels of the hs-CRP. This finding is consistent with results of some of the previous studies (21).

The World Health Organization (WHO) has defined cardiac rehabilitation as the “sum of activity and interventions required to ensure the best possible physical, mental and social conditions so that patients with chronic or post-acute cardiovascular disease may, by their own efforts, preserve or resume their proper place in society and lead an active life” (22).

Cardiac rehabilitation programs, particularly home-based approaches, are well evidenced for secondary prevention in cardiovascular patients. To allow intervention optimization, clinical trials need to apply theory throughout the design, implementation and evaluation stages of the intervention development (23). There is growing evidence that hs-CRP is an important marker of cardiovascular risk and is linked to the pathophysiology of atherosclerosis, providing additional value in primary and secondary prevention. Despite the limitations to its use in routine clinical practice, particularly interindividual variability, the available data indicate that selective determination of hs-CRP is useful in individuals with intermediate cardiovascular risk (10-20% risk at 10 years) in order to optimize risk stratification and clinical management.

A meta regression analysis of nearly 82,000 patients that compared clinical outcomes of lowering LDL-C levels from 10 statin trials versus 9 nonstatin trials showed a 1:1 relationship between LDL-C lowering and CHD and stroke reduction during 5 years of treatment (24). This challenges the idea that pleiotropic effects of statins contribute additional CV risk reduction benefit beyond that expected from the degree of LDL-C lowering. Indeed, some evidence suggests that the previously described proatherogenic effects of CRP may have been overstated because of contamination from endotoxins and use of preservatives in commercial CRP assays (25). Additionally, some basic science research disputes the direct atherogenic effects of CRP. Transgenic over expression of CRP in mice and in vivo injection of large doses of human CRP have minimal effect on inflammation and atherosclerosis (26-30).

Age, recent MI and intra-operative transfusion of red blood cells were also risk factors of peri-operative myocardial infarction and low cardiac output syndrome after cardiac surgery. Patients, suffered from cardiovascular events post-operatively, were older, higher number of patients had recent MI and received donated RBCs intra-operatively but the transfusion of red blood cells remained and independent risk factor of combined cardiovascular event after CABG surgery (31).

Circulating levels of CRP have been shown to increase in patients suffering from acute MI or chronic heart failure (32). Others have found elevated CRP levels among obese or diabetic patients, and correlation of CRP with age (33). In another study, transgenic rabbits with low and high CRP expression fed a high-cholesterol diet experienced similar coronary and aortic atherosclerosis (34).

Long-term decline in CRP is also observed in obese patients (35). Therefore one of important benefits of rehabilitation program in reducing hs-CPR, is its possible impact on the fat tissue. Previous studies have shown that weight loss was significantly associated with a reduction in CRP levels (36). Okita *et al*., have studied 199 healthy women who participated in the two-month period of exercise and have lose three kilograms of their weight (37). In this study, the waist size and body mass index were the most important predictors of CRP levels, indicating a role in accelerating the process of inflammation in obese individuals. However, other aspects of cardiac rehabilitation such as nutrition advice and planning to continue training after the completion of rehabilitation can be helpful in improving the inflammatory processes in these patients. interestingly, a prospective study which aimed to evaluate the effects of exercise on men showed that regular exercise over a period of nine months is associated with a significant reduction in median CRP (38). These studies show that the various components of the rehabilitation program help in decrease in the levels of CRP.

Other studies have focused on the role of genetic factors in the development of inflammatory process. Obisesan *et al*, have studied some of CRP gene variations in relation with exercise and concluded that inhibition of some gene variations that are associated with the CRP is correlated with regular exercise (39). The study showed that patients homozygous for the common haplotype A / G-732 / + 219 have the highest baseline levels of CRP before start of an exercise program. However, CRP genotypes and haplotypes that were examined in this study had no effect on changes in CRP level that is related to exercise.

It is noteworthy that in 2010, a study by Hemingway *et al*. evaluating the quality of research into the prognostic value of CRP in stable CAD. Researchers concluded that about 83 of studies that have been analyzed suffered from a variety of biases and therefor any link between hs-CRP and prognosis is so weak that it cannot be used as the basis for clinical recommendations (40).

## CONCLUSION

In summary, it can be concluded that cardiac rehabilitation might be associated with a significant reduction in serum levels of hs-CRP. Part of this positive effect could be related to weight loss that happens in the interim of rehabilitation program. To prove this connection, it is suggested to conduct a study that specifically evaluates the relationship between reduction of obesity (visceral fat reduction) following cardiac rehabilitation and hs-CRP level.
